# Oral Manifestations of Mucormycosis: A Systematic Review

**DOI:** 10.3390/jof9090935

**Published:** 2023-09-16

**Authors:** Alejandro Mora-Martínez, Laura Murcia, Francisco Javier Rodríguez-Lozano

**Affiliations:** 1 Department of Special Care in Dentistry, Hospital Morales Meseguer, IMIB-Arrixaca, University of Murcia, 30008 Murcia, Spain; alejandro.moram@um.es (A.M.-M.); fcojavier@um.es (F.J.R.-L.); 2 Department of Health Sciences, Catholic University San Antonio of Murcia, 30107 Murcia, Spain

**Keywords:** mucormycosis, oral manifestations, systematic review

## Abstract

Mucormycosis is a rare, opportunistic, and emerging fungal infection that can rapidly develop into a severe, highly fatal clinical picture. In most cases, it is caused by fungi of the order Mucorales, which are usually avirulent but become pathogenic when the host’s immune system is compromised. This systematic review was conducted according to PRISMA guidelines. The databases searched included PubMed, Scopus, and Web of Science. We chose articles that analyzed the oral manifestations of patients with mucormycosis, were published between 2018 and 2023, and met our search terms. The risk of bias in the articles was assessed using the CARE guideline for case reports and STROBE for a cross-sectional study. After the selection process, 20 articles were included in this review, all containing information about the different oral manifestations presented by people with mucormycosis. The most common oral manifestations are mainly bone exposures and oral ulcers, halitosis, pus discharge, gingival thickening, and periodontitis. However, despite the importance of recognizing these oral manifestations in the early stages of mucormycotic infection, providing early treatment, and reducing the high mortality rate of the infection, more studies are needed.

## 1. Introduction

Mucormycosis, also known as zygomycosis, was first described in humans in 1885 by the German pathologist Paultauf [1]. It is a rare, opportunistic, and emerging fungal infection that can rapidly develop into a severe, highly fatal clinical picture [2,3].

In most cases, it is caused by the fungus *Rhizopus oryzae*, of the order *Mucorales* [2]. It is a saprophytic fungus found in soil, animal manure, decaying vegetation, or bread mold [4]. Its mode of transmission is based on the transport of asexual fungal spores through the air; three modes of transmission can be distinguished: inhalation, ingestion, and percutaneous introduction [5]. These fungi are usually avirulent and only become pathogenic when the host’s immunity is significantly reduced [1].

The incidence varies according to geographical area and study period. The highest number of cases has been reported in India, with up to 140 cases per million inhabitants [6,7], likely due to the high endemicity of uncontrolled diabetes and the humid climate [8]. In Europe, the incidence is lower, and the predominant genus is *Lichtheimia* [7].

Thus, there are many predisposing factors, such as renal failure, liver failure, prolonged treatment with immunosuppressive therapy, leukemia, organ transplants, polytrauma, AIDS, or tuberculosis. However, the main factor is uncontrolled diabetes, which is present in 60–80% of mucormycosis cases [5]. Hyperglycemia coupled with low serum pH (<7′35) affects the phagocytic effect of macrophages and the chemotactic and oxidative response of neutrophils, thus decreasing host defense against mucormycosis [9].

Currently, as the number of people undergoing treatment with chemotherapy or immunotherapy increases, this factor is becoming more important [10]. Furthermore, it has also been observed that people with a history of severe COVID-19 are more vulnerable to infection [9].

**Figure 1:**
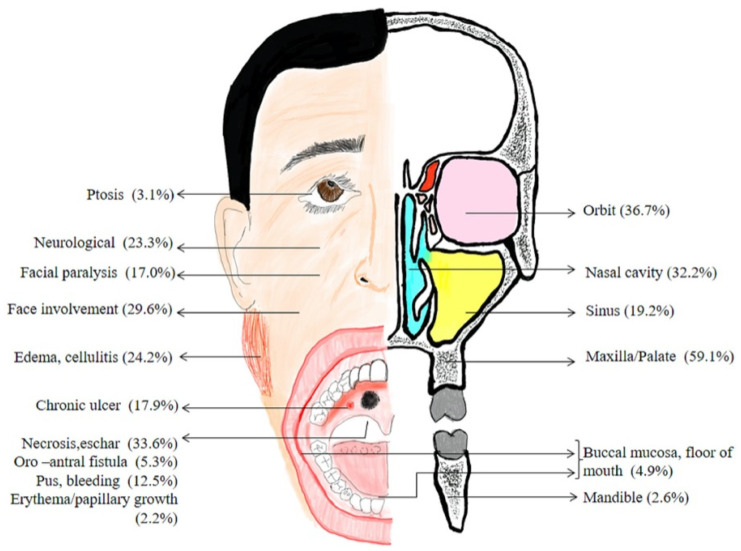
Location of involvement and clinical features of rhinocerebral mucormycosis from article by Kumar et al. [3].

Depending on its clinical forms, mucormycosis can be classified into five types: rhinocerebral, pulmonary, cutaneous, gastrointestinal, and disseminated. The rhinocerebral form is the most common, as well as the most important for dentists, due to the oral manifestations it can present (Figure 1). These are mainly caused by the direct spread of the infection from the sinus to the hard palate, thus causing sudden tooth mobility, perforation of the hard palate, pus secretion, painful necrotic ulcerations, gingival thickening, and halitosis [3,9].

Common primary extra-oral signs of infection include headache, sinus pain, congestion, and bloody nasal discharge [9]. Other signs can also appear, such as fever, facial pain and swelling, cephalea, and trigeminal and facial nerve paralysis [3].

Mucormycosis has a rapid progression and an incubation period that is not well established, depending on the patient’s risk factors; therefore, rapid recognition of signs and symptoms is crucial. In first few days, fever, decreased visual acuity, and facial edema will appear. After 1–2 weeks, bone exposure and oral ulcers can be observed in some cases [5,9,11]. Thus, oral symptoms can already be observed in the early stages of the infection [2].

**Figure 2:**
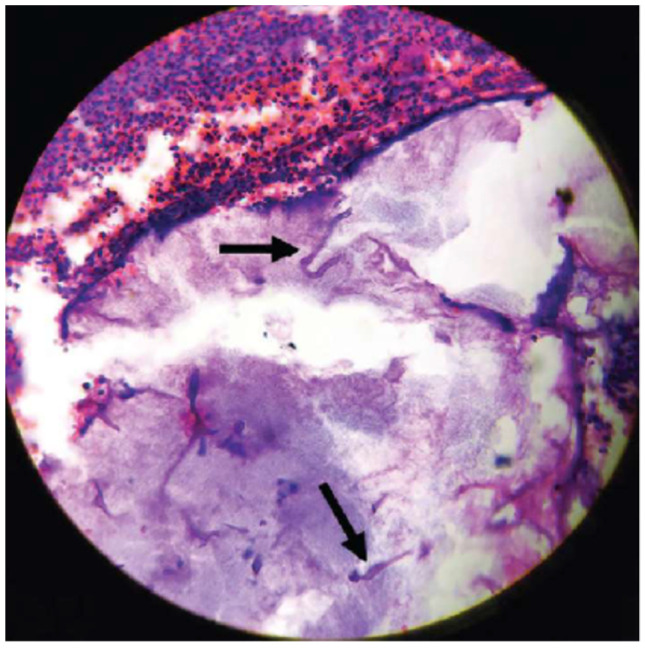
Microscopic observation of non-septate fungal hyphae of mucormycosis noted on H-E staining at ×40 from the article by Deshpande et al. [12].

However, these clinical signs cannot provide a definitive diagnosis of mucormycosis; therefore, a histological diagnosis is needed, where mucorales appear as hyaline filaments in the form of strips and hyphae with variable diameters [5,6]. Usually it can be seen in the hematoxylin-eosin stain (H-E) (Figure 2), but if there are fewer samples or its concentration in the sample is low, calcofluor white is used, which makes it easier to observe [6]. Even so, there are cases where the diagnosis is unclear, and polymerase chain reaction (PCR) is used to detect fungal DNA up to 3 days earlier than histopathological diagnosis [3].

There are four critical factors for adequate treatment: early diagnosis, control of underlying predisposing factors, extensive surgical debridement with margins of healthy tissue, and appropriate antifungal therapy [2]. The main medication used is liposomal amphotericin B (5–10 mg/kg/day), which has a high nephrotoxic potential. Regular monitoring of the patient’s renal function is also necessary. The duration of antifungal treatment can sometimes be extended to months. Since decisions are made based on the patient’s condition, therapy is continued until all clinical and radiological signs are resolved [9,10].

Once the risk factors are under control and successful surgical and pharmacological treatment is completed, the patient’s oral rehabilitation begins via free flaps or by constructing prosthetic devices [13].

Currently, research has focused on adjuvant therapies, especially modulating the tissue microenvironment to deter fungus and enhance the host’s immune response [7].

Nowadays, mucormycosis is an infection with increasing incidence. Its rhinocerebral form is the most common and usually presents oral manifestations. Knowledge of these manifestations facilitates early diagnosis and allows the initiation of treatment immediately, thus increasing the possibility of survival. Therefore, knowing the oral manifestations associated with mucormycosis and conducting a systematic review is essential.

The main objective of this systematic review was to conduct a qualitative synthesis of studies referring to the oral manifestations of mucormycosis.

## 2. Materials and Methods

This systematic review was conducted according to the PRISMA 2020 guidelines, an acronym for “Preferred Reporting Items for Systematic reviews and Meta-Analyses”. It was designed to help authors document their reasons for conducting the review, how they conducted it and what was found during the review. These guidelines help authors conduct a more adequate, structured, and systematic review [14]. Additionally, our review was accepted into the PROSPERO registry (number CRD42022377950), which is an international database of systematic health-related reviews that avoids duplication of similar reviews.

To guide this systematic review, we followed the PICO method, which is used to conduct correct searches for scientific information. Hence, our research question was: What are the oral manifestations of patients with mucormycosis (P: patients with mucormycosis; I: -; C: healthy patients; O: oral manifestations of patients with mucormycosis).

### 2.1. Inclusion Criteria

Articles were included or excluded according to the criteria shown in Table 1.

### 2.2. Search Strategy

#### 2.2.1. Databases

We conducted an exhaustive search was to identify and analyze articles containing relevant information for our systematic review. The following databases were utilized: PubMed, Scopus, and Web of Science. The initial search was conducted on 15 November 2022, and the updated search was performed on 3 February 2023.

#### 2.2.2. Search Terms

The terms used for our search were: “fungal infection”, “mucormycosis”, “dental”, “oral manifestation*”, and “oral disease”. Boolean operators (“AND” and “OR”) were employed to establish relationships between these terms. Table 2 contains the results obtained from each database.

#### 2.2.3. Study Selection

We exported the results to the EndNote bibliographic manager (Clarivate Analytics) after the bibliographic search. First, we eliminated duplicate articles; then, based on the title and abstract, we exclude articles that did not meet our inclusion and exclusion criteria. In cases where information was inconclusive, the full text was read and analyzed to determine eligibility.

#### 2.2.4. Data Extraction

For data extraction, the following categories were considered in each article: author, year of publication, type of study, medical history, dental history, and the most prevalent oral manifestations and localizations. Other data were also extracted, such as country, number of patients in each study, gender, and age.

### 2.3. Quality Evaluation

We followed two different guidelines to analyze the quality of the articles selected for this systematic review: the ‘CAse Reports’ (CARE) and ‘Strengthening the Reporting of Observational Studies in Epidemiology’ (STROBE).

The CARE guideline, consisting of a checklist with 13 items [15], was used to analyze the quality of case reports. To classify cases according to bias risk, the following groups were established according to the percentage of items that comply: ≥70% low risk of bias, 69–50% moderate risk of bias, and ≤49% high risk of bias.

We used the STROBE guide to conduct a cross-sectional study, which is based on 22 points related to different parts of the articles [16]. The articles were classified according to the points they met to determine study bias: low risk (16–22), moderate risk (8–15) and high risk (≤7).

Finally, each point assessed by the guidelines was marked with a tick (✓) if the requirement was met and a cross (✕) if it was not met.

## 3. Results

### 3.1. Study Selection and Flow Diagram

We conducted an exhaustive search of the databases, identifying 953 references related to the oral manifestations of mucormycosis. Of these, 406 were from PubMed, 411 from Scopus, and 136 from Web of Science. Then, we used the bibliographic manager EndNote to remove 241 duplicate articles. A total of 712 articles were analyzed based on their titles and abstracts. However, 678 articles did not meet the inclusion criteria and were excluded.

**Figure 3:**
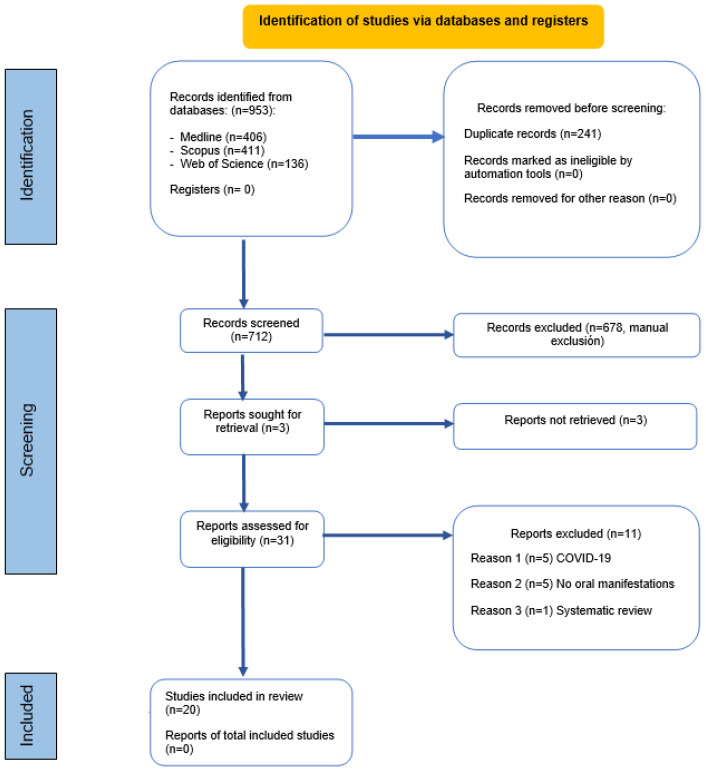
Systemic flow diagram representing the study inclusion process according to the PRISMA 2020 criteria.

Three articles met the criteria and were requested for retrieval but could not be obtained. As a result, only 31 articles were read in full text. Among these, five included cases of COVID-19 that were not mentioned in the title or abstract, five did not present oral manifestations, and only one was a systematic review that was consequently discarded. Finally, 20 articles were selected for analysis (Figure 3).

### 3.2. Results of Data Extraction

Table 3 and Table 4 present the data extraction results from the articles, where the categories above are analyzed.

Most articles presented diabetes as the main medical history associated with mucormycosis, except for four articles. The dental history associated with mucormycosis mainly included a history of previous extractions and previous or recent cases of periodontitis.

**Figure 4:**
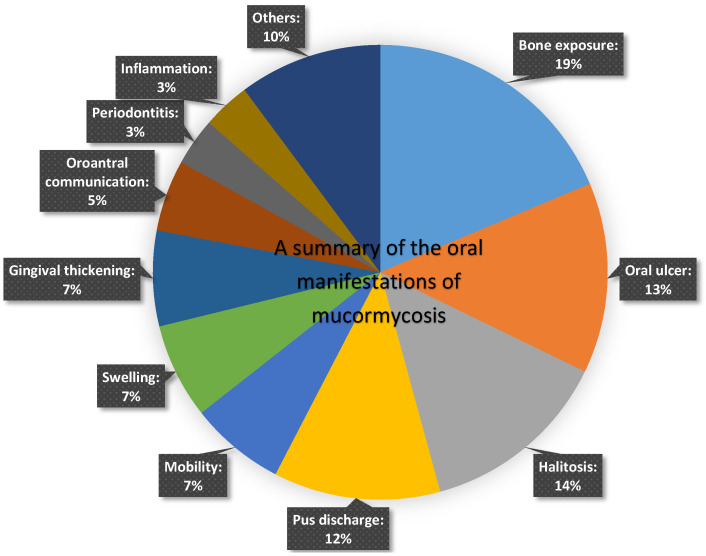
A summary of the oral manifestations of mucormycosis.

All the analyzed articles reported oral manifestations associated with mucormycosis. Oral ulcers and areas of exposed bone, often associated with necrosis, were the most common manifestations. Figure 4 presents a summary of the oral manifestations of mucormycosis.

These lesions were predominantly located in the maxilla, affecting the hard palate and the alveolar ridge. Only one atypical case involving the mandible was observed in this review.

### 3.3. Quality Evaluation

Table 5 and Table 6 present the articles’ quality analysis results. Most of the publications evaluated were case reports, but there was also a cross-sectional study and a prospective case series analysis. Therefore, two guidelines were used for quality analysis: CARE for case reports and case series, and STROBE for cross-sectional studies.

**Figure 5:**
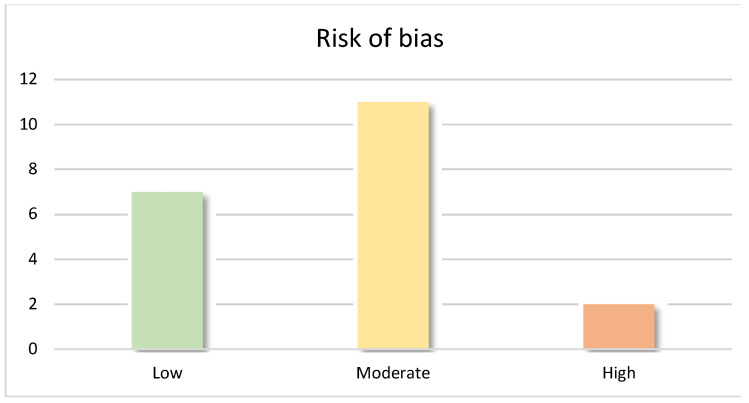
Distribution of articles according to the risk of bias.

The analysis revealed a moderate–low risk of bias among the articles, with 11 studies showing moderate bias and 7 with low risk; only two articles were found to have a high risk of bias (Figure 5).

Following the quality analysis, we decided to exclude two articles with a high risk of bias from the discussion of results to avoid affecting the quality of our findings.

A relevant aspect of our review is that all case reports fulfilled items 5 and 6, which refer to patient information and clinical findings. Only two articles [17,27] did not fulfill item 5d, which discusses concomitant diseases or previous interventions. Additionally, it is worth noting that all cases met items 8a and 8c related to the diagnostic method and its corresponding reasoning.

Only item 12 was addressed by all articles, as none included the patient’s perspective or experience.

Finally, not all case reports provided information on informed consent from patients [18,21,24,35].

### 3.4. Bibliometric Analysis

The articles were distributed according to year of publication, country of publication, journal of publication, and type of article.

**Figure 6:**
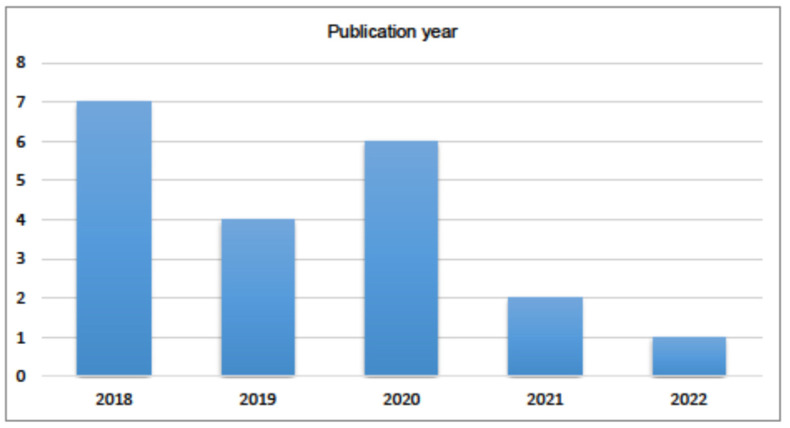
Distribution of articles by year of publication.

Regarding the year of publication (Figure 6), the peak of published articles was in 2018 with seven publications, followed by 2020 with six articles. However, there was a significant decrease in the number of articles in the subsequent years, with only one publication found in 2022. This decline may be directly related to the emergence of COVID-19, as most mucormycosis cases reported during that period were associated with Coronavirus antecedents.

**Figure 7:**
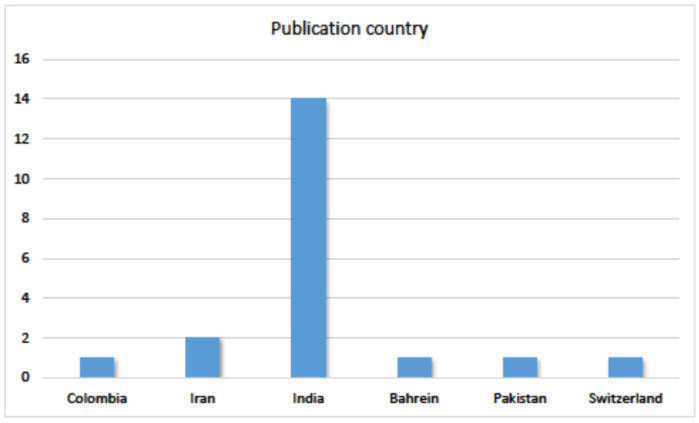
Distribution of articles by country of publication.

Regarding countries of publication (Figure 7), there was a notable dominance of studies published in India, with 14 articles. Only one publication per country was found in all other countries except Iran, where two studies were published.

**Figure 8:**
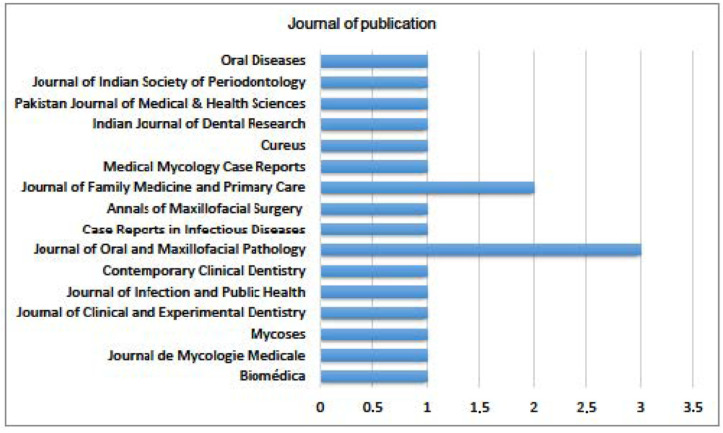
Distribution of articles by journal of publication.

Regarding published journals (Figure 8), the analysis was quite heterogeneous, with only two journals having more than one publication: the Journal of Oral and Maxillofacial Pathology had three studies, and the Journal of Family Medicine and Primary Care had two articles.

**Figure 9:**
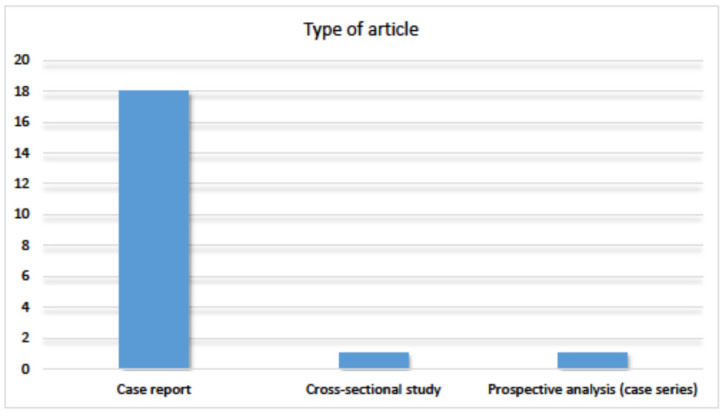
Distribution of articles by type of article published.

Concerning the type of article published (Figure 9), case reports were the most common, totaling 18. In addition, one cross-sectional study and one prospective analysis of a case series were found.

## 4. Discussion

Mucormycosis is an opportunistic fungal infection that progresses rapidly if not diagnosed early and treated immediately [3]. The incidence of mucormycosis has increased in recent years, leading to an increase in studies on mucormycosis and guidelines for diagnosis and treatment [10]. Therefore, as health professionals, dentists must be qualified to recognize and differentiate the signs that appear in the oral cavity.

We analyzed twenty articles that met the inclusion criteria in this review. After assessing their quality, two articles were excluded from the discussion due to a high risk of bias.

As observed, mucormycosis, mainly in its rhinocerebral form, clearly affects the orofacial region. Therefore, knowing the oral manifestations that patients may present is crucial.

In the cross-sectional study conducted by Nezafati et al. between 2007 and 2017 in an Iranian hospital, 40 patients with rhinocerebral mucormycosis were identified, with 72.5% exhibiting oral manifestations. Palatal necrosis was the most frequent manifestation, followed by palatal ulcers, aphthous ulcers, and tongue lesions. Another important finding was that seven of the cases evaluated in the study developed rhinocerebral mucormycosis after tooth extraction, and all had diabetes as a predisposing factor [19]. Thus, the high vascularization of the maxilla, combined with a compromised immune response, appears to be closely associated with post-extraction mucormycosis.

Gholinejad Ghadi et al., Prabhu et al., Rai et al., Rani et al., Pandilwar et al., and Rajashri et al. reported cases of mucormycosis in patients with uncontrolled diabetes and a history of tooth extraction, suggesting that tooth extraction is an important dental history to consider, because it participates in the development of mucormycosis, especially in diabetic patients [18,21,22,26,29,31].

Atypical cases have also been identified, such as those reported by Nilesh et al. and Venkatesh et al., where immunocompetent patients developed fungal infections after tooth extraction. Consequently, the extraction of teeth, particularly maxillary molars, may increase a patient’s susceptibility to mucormycosis due to the proximity of the maxillary sinus, which is often affected by inhalation of the spores [20,23].

In the reported cases by Rai et al., Venkatesh et al., and Deshpande et al., cases of periodontitis have been found to act as a dental antecedent of mucormycosis. Although not the only antecedent, periodontal disease was associated with tooth extractions in all three cases. Therefore, patients with periodontitis should follow a strict periodontal protocol to prevent tooth extraction since, as mentioned previously, the extraction of teeth, especially maxillary molars, increases susceptibility to mucormycosis [12,22,23].

In addition to dental history, Beiglboeck et al. reported a case of mucormycosis in which the patient had undergone molar endodontic treatment a week before, which may have caused or at least favored the fungal infection [35].

Only cases published by Ramadorai et al., Srivastava et al. Ramesh et al., Verma et al., and Anwar et al. had no dental history highlighted [25,27,32,33,34]. Thus, as seen in most cases, dental history generally plays a vital role in the development of rhinocerebral mucormycosis, which can increase susceptibility to its progression.

In terms of medical history, more than half of the cases analyzed had a history of uncontrolled diabetes. Only Panneerselvam et al. and Verma et al. reported mucormycosis in patients with controlled diabetes [30,33]. Only two cases published by Srivastava et al. and Agarwal et al. were unrelated to diabetes. The only medical history the authors reported was trauma to the cheekbone and the presence of chronic granulomatous disease, respectively [27,28].

Thus, as mentioned by Anwar et al., diabetes interferes with the body’s immune response to infection. A high glycemic index increases fungal proliferation and reduces chemotaxis and phagocytic efficacy [34].

Our next step is to analyze its oral manifestations, having described the medical and dental history associated with several cases of mucormycosis.

In their prospective analysis, Ramadorai et al. presented a series of ten case reports, all with uncontrolled diabetes, where swelling in the maxillary region around the face was the main symptom. Intraorally, the two most frequent oral manifestations of mucormycosis could be observed: palatal ulcers and areas of bone exposure. Only one patient showed signs of oroantral communication, indicating an advanced stage of mucormycosis [25]. This article is in line with Nezafati et al.’s study, where swelling was the most frequent sign during the presentation of infection, together with palatal ulcers and bone involvement, which were the two most frequent oral manifestations [19]. Therefore, facial swelling and pain signs precede oral ulcers, which can progress to necrotic bone exposure and palatal perforations [12,23,29].

Some articles, such as those by Rai et al. and Deshpande et al., indicate oral ulceration as the pathognomonic lesion typical of rhinocerebral mucormycosis, usually followed by necrotic bone exposure [12,22]. However, in the present review, the most frequent oral manifestation was bone exposure, with eleven publications presenting bone exposure compared to seven publications reporting oral ulcers. This frequency suggests that mucormycotic infection was at an advanced stage in these cases. Furthermore, in cases published by Gholinejad Ghadi et al., Rai et al., Rani et al., Srivastava et al., Agarwal et al., and Pandilwar et al., it is evident that bone lesions are associated with necrosis in most cases, exacerbating the situation [18,22,26,27,28,29].

Apart from prevalent manifestations, there are also less frequent manifestations, such as halitosis, pus secretion, gingival thickening, or periodontitis.

Halitosis and pus secretion are usually associated with bone exposures and oral ulcers, which are the most frequent manifestations [18,20,26,27,28,29,30]. Similarly, patients with gingival thickening have also presented with halitosis and pus discharge [28,30].

Another atypical manifestation worth considering is periodontitis, which can also be a predisposing factor for developing mucormycosis. Deshpande et al. reported an infrequent case where mucormycosis simulated severe periodontitis, causing delayed diagnosis and a worse prognosis. Thus, although the main oral manifestations of mucormycosis are ulcers and bone exposures, other non-specific presentation forms may delay diagnosis, such as warty lesions, painful indurated ulcers, or periodontal signs [12].

In terms of the locations of oral manifestations, most cases in this review involved the hard palate, followed by the alveolar ridge and the alveolar bone. These results agree with Venkatesh et al., Ramesh et al., and Verma et al., who established the palate as the most frequent oral location for these manifestations [23,32,33].

However, Rai et al. showed less frequent locations of involvement, such as the gums, cheeks, tongue, and jaw [22], which were also presented in case reports by Gholinejad Ghadi et al., Nezafati et al., Ramadorai et al., and Agarwal et al. [18,19,25,28].

In this review, practically all of the articles were case reports, which had a relatively low level of scientific evidence. However, they were included because no articles with a high level of evidence met the established inclusion criteria for this review. Another relevant aspect is that most articles focused on the infection’s systemic manifestations. In other words, information about oral manifestations was scarce.

As observed throughout this systematic review, knowledge of oral manifestations, which often appear in patients with mucormycosis in its rhinocerebral form, can aid in early diagnosis and improve patient survival with immediate treatment. This review also demonstrates how certain dental histories can increase the likelihood of developing this infection. Dentists and other healthcare professionals should be mindful of these factors to mitigate complications and reduce mucormycosis mortality rates.

## 5. Conclusions

Mucormycosis is an opportunistic fungal infection that can rapidly progress when the host’s immunity is compromised. The rhinocerebral form mainly affects the oral cavity, since the maxillary sinus and hard palate can be directly infected when spores are inhaled nasally.

Mucormycosis development is more likely in individuals with specific medical and dental antecedents that compromise their immune status, particularly uncontrolled diabetes. Nevertheless, previous histories of tooth extraction and, less commonly, periodontitis and endodontic procedures have also been associated with an increased risk of mucormycosis.

The most common oral manifestations are mainly bone exposures and oral ulcers, halitosis, pus discharge, gingival thickening, and periodontitis.

However, despite the importance of recognizing oral manifestations in the early stages of mucormycotic infection to initiate immediate treatment and reduce the high mortality rate of the infection, there are still scarce studies on these manifestations.

## Figures and Tables

**Table 1:** Inclusion and exclusion criteria.

INCLUSION CRITERIA	EXCLUSION CRITERIA
Published between 2018 and 2023	Articles without full text availability
Articles in English or Spanish	Published in a language other than English or Spanish
Articles of free full text access or available through the University of Murcia	Articles written exclusively about systemic manifestations of mucormycosis
With analyzed results obtained	Cases of mucormycosis associated with COVID-19
Information about oral manifestations in patients with mucormycosis	
Followed our search terms	

**Table 2:** Findings of each database.

Database	Search Strategy	Results
Medline (PubMed)	1# “fungal infection” OR mucormycosis	19,209
	2# dental OR “oral manifestation*” OR “oral disease”	664,949
	1# AND 2#	406
Scopus	1# “fungal infection” OR mucormycosis	44,271
	2# dental OR “oral manifestation*” OR “oral disease”	591,253
	1# AND 2#	411
Web of Science	1# “fungal infection” OR mucormycosis	20,253
	2# dental OR “oral manifestation*” OR “oral disease”	235,769
	1# AND 2#	136

**Table 3:** Description of the differentiated variables for each examined article.

Author, Publication Year	Type of Study	Medical History	Dental History	Most Prevalent Oral Manifestations	Most Frequent Locations
Bravo et al. [17], 2018	Case report	Non-controlled diabetes	Extraction	Periodontal abscess, oral ulcer, and oroantral communication	Alveolar ridge and hard palate
Gholinejad Ghadi et al. [18], 2018	Case report	Non-controlled diabetes, neutropenia, and ischemic cardiopathy	Extraction	Swelling, gingival thickening, and necrotic bone exposure	Maxilla, gums, alveolar ridge, and hard palate
Nezafati et al. [19], 2018	Cross-sectional study	Diabetes, trauma, MDS, asthma, arthritis rheumatoid, radiotherapy, chemotherapy, and glomerulonephritis	Extractions	Necrosis, oral ulcers, aphthous, and inflammation	Palate, buccal mucosa, and tongue
Nilesh et al. [20], 2018	Case report	Healthy	Extractions	Oroantral fistula, necrosis, bone exposure, and halitosis	Maxilla, alveolar bone, and buccal mucosa
Prabhu et al. [21], 2018	Case report	Non-controlled diabetes	Extraction	Necrosis	Hard palate, alveolar ridge, and buccal mucosa
Rai et al. [22], 2018	Case report	Non-controlled diabetes	Severe periodontitis and extractions	Necrotic oral ulcer, periodontitis, and necrotic bone exposure	Hard palate and alveolar bone
Venkatesh et al. [23], 2018	Case report	Healthy	Generalized periodontitis and extractions	Bone exposure and halitosis	Maxilla
Arani et al. [24], 2019	Case report	Controlled diabetes	Extraction	Swelling, oroantral communication, and pus discharge	Alveolar ridge and maxillary sinus floor
Ramadorai et al. [25], 2019	Prospective analysis (case series)	Non-controlled diabetes and bronchial asthma	None	Swelling, oral ulcer, bone exposure, bone sequestration, and oroantral communication	Palate, cheek, and alveolar bone
Rani et al. [26], 2019	Case report	Non-controlled diabetes and controlled hypertension	Extraction	Erosion, necrotic bone exposure, and halitosis	Hard palate and maxillary arch
Srivastava et al. [27], 2019	Case report	Trauma to the cheekbone	None	Swelling, pus discharge, abscess, necrotic bone exposure, and mobility	Alveolar ridge and hard palate
Agarwal et al. [28], 2020	Case report	Chronic granulomatous disease	Extractions	Mobility, gingival thickening, necrotic bone exposure, pus discharge, and halitosis	Jaw and gums
Pandilwar et al. [29], 2020	Case report	Non-controlled diabetes	Extraction	Necrotic bone exposure, necrotic oral ulcer, mobility, pus discharge, and halitosis	Alveolar bone and hard palate
Panneerselvam et al. [30], 2020	Case report	Controlled diabetes	Extractions and curettage	Gingival thickening and pus discharge	Alveolar ridge and hard palate
Rajashri et al. [31], 2020	Case report	Non-controlled diabetes	Extraction	Necrosis, bone exposure, and halitosis	Maxillary alveolar bone and buccal mucosa
Ramesh et al. [32], 2020	Case report	Non-controlled diabetes and dengue	None	Oral ulcer, bone exposure, and halitosis	Hard palate
Verma et al. [33], 2020	Case report	Controlled diabetes	None	Necrotic oral ulcer, pus discharge, and halitosis	Hard and soft palate
Anwar et al. [34], 2021	Case report	Non-controlled diabetes	None	Necrotic oral ulcer and pus discharge	Hard palate
Deshpande et al. [12], 2021	Case report	Non-controlled diabetes and hypertension	Periodontitis, extractions, and endodontics	Gingival thickening, mobility, and periodontal pockets	Alveolar ridge and maxilla
Beiglboeck et al. [35], 2022	Case report	Non-controlled diabetes, cirrhosis, kidney disease, and hypertensive heart disease	Endodontics	Inflammation	Maxilla and alveolar ridge

**Table 4:** Description of the other differentiated variables for each examined article.

	Country	Number of Patients in Each Study	Gender	Age
Bravo et al. [17]	Colombia	1	Male	63
Gholinejad Ghadi et al. [18]	Iran	2	Female Male	36 53
Nezafati et al. [19]	Iran	40	Female 19 Male 21	Average age of 60.6
Nilesh et al. [20]	India	2	Male Male	52 37
Prabhu et al. [21]	Bahrein	1	Male	70
Rai et al. [22]	India	1	Male	57
Venkatesh et al. [23]	India	1	Male	32
Arani et al. [24]	India	1	Male	48
Ramadorai et al. [25]	India	10	3 Female 7 Male	Average age of 49.4
Rani et al. [26]	India	1	Male	63
Srivastava et al. [27]	India	1	Male	42
Agarwal et al. [28]	India	1	Male	37
Pandilwar et al. [29]	India	2	Male Male	60 67
Panneerselvam et al. [30]	India	1	Female	45
Rajashri et al. [31]	India	1	Male	55
Ramesh et al. [32]	India	1	Male	23
Verma et al. [33]	India	1	Female	58
Anwar et al. [34]	Pakistan	1	Female	50
Deshpande et al. [12]	India	1	Female	46
Beiglboeck et al. [35]	Switzerland	1	Male	74

**Table 5:** Quality assessment of the cross-sectional study using the STROBE guide.

	1	2	3	4	5	6	7	8	9	10	11	12	13	14	15	16	17	18	19	20	21	22	Total Score	Risk of Bias *
Nezafati et al. (2018) [19]	✓	✓	✓	✓	✓	✓	✕	✓	✕	✓	✕	✓	✓	✓	✓	✕	✕	✓	✓	✓	✓	✓	17	Low

*Items*: 1—Title and abstract, 2—Background/rationale, 3—Objectives, 4—Study design, 5—Setting, 6—Participants, 7—Variables, 8—Data sources/measurement, 9—Bias, 10—Study size, 11—Quantitative variables, 12—Statistical methods, 13—Participants, 14—Describe data, 15—Outcome data, 16—Main results, 17—Other analyses, 18—Key results, 19—Limitations, 20—Interpretation, 21—Generalisability and 22—Funding. * Risk of bias: low risk (16–22), moderate risk (8–15) and high risk (≤7).

**Table 6:** Quality assessment of case reports using the CARE guide.

	1	2	3				4	5				6	7	8				9			10				11				12	13	Risk of Bias * [%]
			a	b	c	d		a	b	c	d			a	b	c	d	a	b	c	a	b	c	d	a	b	c	d			
Bravo et al. [17]	✓	✓	✕	✕	✕	✕	✕	✓	✓	✓	✕	✓	✕	✓	✓	✓	✕	✓	✓	✕	✕	✕	✕	✓	✕	✓	✕	✕	✕	✓	High [46.67%]
Gholinejad Ghadi et al. [18]	✓	✓	✕	✓	✓	✓	✓	✓	✓	✓	✓	✓	✓	✓	✕	✓	✓	✓	✓	✓	✕	✓	✓	✓	✓	✓	✓	✓	✕	✕	Low [83.33%]
Nilesh et al. [20]	✓	✕	✕	✓	✓	✕	✓	✓	✓	✓	✓	✓	✓	✓	✕	✓	✓	✓	✓	✕	✕	✓	✓	✕	✕	✓	✓	✕	✕	✓	Moderate [66.67%]
Prabhu et al. [21]	✕	✕	✓	✓	✓	✕	✕	✓	✓	✓	✓	✓	✕	✓	✕	✓	✕	✓	✓	✕	✕	✓	✓	✓	✓	✓	✓	✓	✕	✕	Moderate [63.33%]
Rai et al. [22]	✓	✓	✕	✓	✕	✓	✓	✓	✓	✓	✓	✓	✓	✓	✕	✓	✓	✓	✓	✓	✓	✓	✕	✓	✕	✓	✓	✓	✕	✓	Low [80%]
Venkatesh et al. [23]	✕	✕	✓	✓	✕	✕	✕	✓	✓	✓	✓	✓	✓	✓	✕	✓	✕	✓	✓	✕	✕	✕	✕	✕	✓	✓	✓	✓	✕	✓	Moderate [56.67%]
Arani et al. [24]	✓	✕	✕	✓	✕	✕	✕	✓	✓	✓	✓	✓	✕	✓	✕	✓	✕	✓	✓	✕	✕	✕	✕	✕	✕	✓	✓	✓	✕	✕	High [46.67%]
Ramadorai et al. [25]	✕	✓	✓	✓	✓	✓	✕	✓	✓	✓	✓	✓	✕	✓	✕	✓	✓	✓	✓	✓	✓	✕	✓	✓	✓	✓	✓	✓	✕	✓	Low [80%]
Rani et al. [26]	✕	✓	✓	✓	✕	✕	✓	✓	✓	✓	✓	✓	✕	✓	✕	✓	✕	✓	✕	✕	✕	✕	✕	✕	✓	✓	✓	✕	✕	✓	Moderate [53.33%]
Srivastava et al. [27]	✕	✓	✕	✓	✓	✕	✓	✓	✓	✓	✕	✓	✓	✓	✕	✓	✕	✓	✓	✕	✕	✕	✓	✓	✕	✓	✓	✓	✕	✓	Moderate [63.33%]
Agarwal et al. [28]	✓	✓	✓	✓	✓	✓	✓	✓	✓	✓	✓	✓	✓	✓	✓	✓	✓	✓	✓	✓	✓	✓	✓	✕	✕	✓	✓	✓	✕	✓	Low [90%]
Pandilwar et al. [29]	✕	✓	✕	✓	✕	✓	✓	✓	✓	✓	✓	✓	✕	✓	✕	✕	✓	✓	✓	✕	✕	✕	✓	✕	✕	✓	✓	✓	✕	✓	Moderate [60%]
Panneerselvam et al. [30]	✕	✓	✓	✓	✓	✓	✕	✓	✓	✓	✓	✓	✕	✓	✕	✓	✓	✓	✕	✓	✕	✓	✓	✓	✓	✓	✓	✓	✕	✓	Low [76.67%]
Rajashri et al. [31]	✓	✕	✕	✓	✕	✕	✕	✓	✓	✓	✓	✓	✕	✓	✕	✓	✓	✓	✓	✕	✓	✓	✕	✕	✕	✓	✕	✓	✕	✓	Moderate [56.67%]
Ramesh et al. [32]	✕	✓	✕	✓	✕	✕	✕	✓	✓	✓	✓	✓	✓	✓	✕	✓	✓	✓	✓	✕	✕	✕	✓	✕	✕	✓	✕	✕	✕	✓	Moderate [53.33%]
Verma et al. [33]	✓	✓	✕	✓	✕	✕	✓	✓	✓	✓	✓	✓	✓	✓	✕	✓	✕	✓	✓	✕	✕	✕	✕	✕	✕	✓	✓	✕	✕	✓	Moderate [56.67%]
Anwar et al. [34]	✓	✓	✕	✓	✓	✕	✓	✓	✓	✓	✓	✓	✕	✓	✕	✓	✓	✓	✕	✓	✓	✕	✕	✓	✕	✓	✕	✓	✕	✓	Moderate [66.67%]
Deshpande et al. [12]	✕	✓	✓	✓	✓	✕	✓	✓	✓	✓	✓	✓	✕	✓	✕	✓	✓	✓	✓	✓	✓	✕	✕	✓	✓	✓	✓	✓	✕	✓	Low [76.67%]
Beiglboeck et al. [35]	✓	✕	✕	✕	✕	✓	✓	✓	✓	✓	✓	✓	✓	✓	✕	✓	✕	✓	✓	✓	✓	✕	✕	✓	✓	✓	✓	✓	✕	✕	Moderate [66.67%]

Items: 1—Title, 2—Keywords, 3—Abstract, 4—Introduction, 5—Patient information, 6—Clinical findings, 7—Timeline, 8—Diagnostic assessment, 9—Therapeutic intervention, 10—Follow up and outcomes. 11—Discussion, 12—Patient perspective and 13—Informed consent. * Risk of bias: low risk [≥70%], moderate risk [69–50%] and high risk [≤49%].

## Data Availability

All analyzed data are included in this paper.
